# Bromide of Ethyl

**Published:** 1880-10

**Authors:** 


### ARTICLE III.

### *Bromide of Ethyl.*

The following paragraphs from Medical Journals, and reports of hospitals, etc., give evidence of the value of this candidate for place among the anæsthetics in use:

"Dr. Lawrence Wolff, of Philadelphia, has recently published a paper on bromide of ethyl as an anæsthetic, which, in view of the recent discussions as to its properties, possesses more than an ordinary degree of interest. In describing the manner of preparing the drug, mention is made of a brown acrid liquid remaining after the bromide is distilled off, and which possesses an unpleasant and pungent odor. Twenty drops of this latter liquid was administered to a rabbit, causing symptoms of irritation of the gastro-intestinal tract, and death in eighteen hours. At the post-mortem, the brain was found to be congested, the abdominal viscera were also congested and irritated, and the odor of the liquid was given off. On the other hand, 30 grains of the pure bromide of ethyl had been given to the same animal, previous to this, and no unpleasant symptoms were produced. Several experiments were made with similar results. In one instance in which the rabbit died while under the influence of the pure bromide of ethyl, the brain was found to be in an anæmic condition, and the odor of the ethyl could not be detected in the organs. W. is inclined to believe that death in Dr. Sims's case was due to the presence of the heavy distillate above mentioned; in support of this view he says that, ethyl bromide being a volatile substance, its odor would not probably be detected forty-one hours after administering it. He does not believe that it is unstable in its composition, as some say, he having failed to separate the bromide from the ethyl, after repeated attempts. The following conclusions are arrived at: rabbits, which are with difficulty anæsthetized, can be ethylized with success and without danger to life. When employed hypodermically the ethyl causes an increase in the number of respirations. No greater toxic effects are produced on the system by its use than would result from the administration of ether or alcohol, so that danger from this source need not be apprehended. Also, that while it cannot be positively stated that pure ethyl bromide is *per se* an absolutely safe anæsthetic, yet it is as safe as ether, and more so than chloroform. He recommends that the drug should have a place in the new pharmacopœia."—*Reprint from American Journal of Pharmacy*, May, 1880.

But a clinical report of Dr. Charles C. F. Gay, of the Buffalo Hospital, says of this article:

"In the case under observation, the new anæsthetic was administered; it required eleven minutes to bring the patient under its influence, and nine drachms were used. Its odor was very disagreeable, its effect was evanescent. Two drachms had previously put the house physician asleep, and during its administration to the patient, the doctor—who appears to be unusually susceptible to the effects of anæsthetics—came near falling asleep.

Another patient, a very strong man, just as he was coming under the influence of the same anæsthetic, became excited, violent, and unmanageable, sprang from the table, and escaped to his bed."

It would seem, however, from the following paragraph that great care should be used in selecting the drug, for "Dr. Carl Jungk, in the *Therapeutic Gazette*, has given the results of analyzing a large number of samples of hydrobromic ether. Among the samples examined were specimens of the ether used by Dr. J. Marion Sims and of that used by Dr. Levis in their fatal cases. The reactions of these demonstrated conclusively their entire unfitness for purposes of inhalation."

The following is an editorial from the *N. Y. Medical Record*, of June 9th, on the subject, has special reference to the experience of French surgeons:

"The properties and value of this new anæsthetic were quite extendedly discussed at a recent meeting of the Paris Surgical Society. Half a dozen gentlemen related their experience, which, on the whole, gave a qualified endorsement of the drug. The same characteristics, good and bad, which have already been noted by American surgeons, were observed by the French, No fatal cases were reported however.

M. Verneuil was struck with the rapid and powerful action of the anæsthetic. One of his patients, a woman, to whom he gave it, was put to sleep almost in an instant. M. Berger was also impressed with its power; a rabbit put under a bell-glass, in which were placed ten grammes (ʒ iiss.) of bromide of ethyl, expired at once. He had never seen so rapid an action in the case of chloroform or ether. These illustrations may be remembered in connection with the general fact, asserted by Dr. Turnbull, that the danger of an anæsthetic is in proportion to its strength of action. We may state here also the fact that a death is reported to have recently occurred in the practice of Dr. Levis while using the bromide. We are not informed, however, that the anæsthetic was the direct or sole cause of the death.

The opinion was expressed, during the discussion referred to, that the bromide of ethyl did not easily produce complete muscular relaxation, and could not be used in operations where such a condition is essential. The opinions, as we have said, while not against its use, were by no means strongly in its favor. This refers to the ether when inhaled. As a local anæsthetic it was spoken of highly. It is the only anæsthetic of this kind which, owing to its non-inflammability, can be safely used in connection with the actual cautery; such was the opinion of M. Micaise. Evidence of its value in this direction has been given by American surgeons, and the point was referred to by Dr. Turnbull, at the recent meeting of the American Medical Association.

It seems that bromide of ethyl is rapidly finding its true place as an anæsthetic, and that place is not a very high one."

---

### ARTICLE IV.

### *Articulating Artificial Teeth.*

